# The small trial problem

**DOI:** 10.1186/s13063-023-07348-3

**Published:** 2023-06-22

**Authors:** Jean Raymond, Tim E. Darsaut, Johanna Eneling, Miguel Chagnon

**Affiliations:** 1 Department of Radiology, Service of Neuroradiology, Centre Hospitalier de L’Université de Montréal (CHUM), Montreal, QC H2X 0C1 Canada; 2 Division of Neurosurgery, Department of Surgery, University of Alberta Hospital, Mackenzie Health Sciences Centre, Edmonton, AB Canada; 3 Department of Mathematics and Statistics, University of Montreal, Montreal, QC Canada

**Keywords:** Trial size, Sample size, Number of patients, Trial methodology, Problems with continuous variables, Dichotomization, Pragmatic trials, Surgery, Placebo-controlled surgical trials

## Abstract

**Background:**

Many randomized trials that aim to assess new or commonly used medical or surgical interventions have been so small that the validity of conclusions becomes questionable.

**Methods:**

We illustrate the small trial problem using the power calculation of five Cochrane-reviewed studies that compared vertebroplasty versus placebo interventions. We discuss some of the reasons why the statistical admonition not to dichotomize continuous variables may not apply to the calculation of the number of patients required for trials to be meaningful.

**Results:**

Placebo–controlled vertebroplasty trials planned to recruit between 23 and 71 patients per group. Four of five studies used the standardized mean difference of a continuous pain variable (centimeters on the visual analog scale (VAS)) to plan implausibly small trials. What is needed is not a mean effect at the population level but a measure of efficacy at the patient level. Clinical practice concerns the care of individual patients that vary in many more respects than the variation around the mean of a single selected variable. The inference from trial to practice concerns the frequency of success of the experimental intervention performed one patient at a time. A comparison of the proportions of patients reaching a certain threshold is a more meaningful method that appropriately requires larger trials.

**Conclusion:**

Most placebo-controlled vertebroplasty trials used comparisons of means of a continuous variable and were consequently very small. Randomized trials should instead be large enough to account for the diversity of future patients and practices. They should offer an evaluation of a clinically meaningful number of interventions performed in various contexts. Implications of this principle are not specific to placebo-controlled surgical trials. Trials designed to inform practice require a per-patient comparison of outcomes and the size of the trial should be planned accordingly.

## Introduction

The number of patients that need to be recruited for a clinical trial to provide meaningful results is a crucial issue in clinical research. The size of the trial has momentous consequences on the conclusions that can be inferred from trial results to clinical practice. Unfortunately, many investigators are inclined, at the planning stages, to reduce the size of the trial in order to render the trial more feasible, only to find that the resultant trial is too small to be meaningful at the time of publication [1]. Trials that assess the efficacy of commonly used interventions (and especially placebo-controlled surgical trials) are notorious for having difficulties with recruitment. They are often very small [2]. Subsequent interpretation of small trials that fail to show a difference between placebo and active surgery frequently leads to clinical controversy [3–6].

There are many ways to reduce the number of patients that need to be recruited in a trial. Most of these design choices (such as including only patients highly likely to benefit from the intervention) are best described as “explanatory” choices [7]. The strategy we wish to examine here is the use of a continuous variable as the primary outcome measure and to compare the means of two groups of patients (i.e., surgery versus placebo) to estimate trial size. Because clinical practice entails caring for individual patients that vary in many more respects than the variation around the mean of a single selected variable, a more appropriate estimate would require a comparison of the proportions of patients reaching a predetermined per-patient primary outcome threshold indicating clinical success or failure. There are other important aspects pertinent to the choice of sample size that we will not discuss, such as the type of control group, the statistical methods used in the analysis of results, or ethical issues, but here we only wish to contrast per-patient and per-population outcome measures. The dichotomization into good or bad outcomes allows estimation of the individual patient’s chance of success. This approach is considered less powerful, because many more trial patients are needed, but it is a more plausible basis on which to make meaningful inferences from trial results to clinical practice. Although categorizing or dichotomizing continuous variables is almost universally condemned by statisticians [8–10], a solution to the small trial problem requires a more nuanced appreciation of the use of categories as outcome measures, because they play key roles, not only in making clinical decisions but also in measuring the results of medical or surgical interventions at the individual patient level.

The objective of this paper is to discuss and contrast two methods of calculating the size of trials: one commonly used method compares the means of continuous variables. This method is exemplified by most placebo-controlled vertebroplasty trials reported in a Cochrane review [11]. The other method compares the frequency of good outcomes at the level of patients. We will try to defend the latter because although less powerful, it recognizes the primacy of individual patient outcomes in medical care and research. While we use placebo-controlled vertebroplasty trials to illustrate the problem, we believe the problem we discuss and the solution we propose can be generalized to all trials designed to inform clinical practice.

## The primary outcome measure and the minimally important change

A clinical trial is an instrument designed to measure the impact of a treatment on patients. The sample size calculation is important to prevent the erroneous claim that “absence of evidence” is “evidence of absence” [12]. In other words, to prevent the claim that the trial showed no difference, while in truth the trial had no power to show an important difference between treatments. Other authors would consider that the trial hypothesis had not been severely tested, a general criterion for any scientific enterprise [13].

The number of patients to be recruited depends on the research question and on one of its essential components, the primary outcome measure, where the comparative effects of the treatments are evaluated. We will not discuss the difficulties of using a subjective outcome such as pain. Our aim is to contrast the use of the means of a continuous variable such as pain scores (or any other quantitative variable), with the use of categories of patients having reached (or not reached) a certain outcome.

To claim that a difference has been shown (or not), investigators need to predetermine the “minimally clinically significant difference ∆” or the “minimally important change” (MIC) [14]. For a certain power (the capacity to show a difference beyond chance findings, say 80% or 90%), allowing a small alpha error (the error of claiming a difference when none exists, say 5%), the number of patients to be recruited will increase with decreasing ∆. While the importance of the notion of a meaningful difference cannot be over-emphasized, it is difficult to precisely define. There have been many methods and recommendations made, but there is no clear consensus on how to choose ∆ or MIC in practice. Each specialized field may have to empirically investigate that crucial matter for each variable. While clinicians may have some intuition regarding the MIC that may make sense at the patient level, no one knows how to choose the MIC at the level of populations. The 2 different ways of defining the MIC can yield trials of widely different sizes. In addition, expert opinions vary widely, between institution, countries, and contexts. The MIC (at the patient level) should be tested for reliability at the intra-subject, intra-rater, and inter-rater levels. Reliable MICs could also be tested for impact on quality of life or on clinical decisions. In the case of back pain (including from vertebral fractures), the MIC that is frequently used is a 30% improvement from baseline for individual patients, and 1–1.5 points on the 0–10 visual analog scale for groups or populations. These values have been arbitrarily chosen by “expert consensus” [14].

The next problem to consider is variability or heterogeneity. Patients always differ from one another for any characteristic under consideration. Patients also react differently to medical or surgical interventions. For any given ∆, the capacity of the trial to show a meaningful difference between treatments and therefore the number of patients to be recruited in the trial will depend on the variability observed within and between the groups to be compared. But the variability of what, exactly? We examine 2 possibilities: (i) the variability concerns patients: the method should then compare the proportions of patients reaching a certain improvement (for example 30% improvement from baseline) or a certain clinical outcome (success or failure); (ii) the variability concerns the variable; the method then compares the “treatment effect size” with the standard deviation or the distribution and dispersion of the variable of interest. The use of this latter option is commonly recommended, because the number of patients to be recruited is smaller and the trial more feasible. This option was chosen for most of the vertebroplasty trials.

## Placebo-controlled vertebroplasty trials

Osteoporotic vertebral compression fractures (OVCFs) are frequent (700,000 patients every year in the USA) [15, 16]. They can be painful enough to keep patients bedridden for weeks. In elderly patients a host of medical complications, such as metabolic disturbances, muscle wasting, bed sores, pneumonia, septicemia, deep venous thrombosis, and pulmonary emboli can lead to poor clinical outcomes, including death. Treatment options include conservative management (bed rest, thoracolumbar braces, various analgesic and anti-osteoporotic drugs and physiotherapy) or more recently minimally invasive procedures such as vertebroplasty or kyphoplasty. The placebo-controlled trials that were designed to test whether vertebroplasty improves patient outcomes have yielded conflicting but mainly disappointing results [11]. While these procedures have been widely used in practice, all of the vertebroplasty trials reported in the Cochrane review were small. Small trials are not unique to vertebroplasty. Most placebo-controlled surgical trials have included fewer than 100 patients (median 61 patients in 53 trials of a systematic review) [2].

Table 1 summarizes the sample size calculations reported for the various placebo-controlled vertebroplasty trials. All of the trials (except the one by Clark et al.) compared the means of a continuous pain scale variable (visual analog scale (VAS)) to yield implausibly small trials (between 23 and 71 patients per group).

**Table 1:** Size calculations of vertebroplasty trials

Author—year	Expected treatment effect (cm in VAS)^a^	Standard deviation	Time of evaluation	Number of patients per group^b^
Buchbinder 2009 [17]	2.5	3.0	3 months	24
Kallmes 2009 [18]	1.5	2.7	1 month	65
Firanescu 2018 [19]	1.5	4.2	Multiple time points	71
Hansen 2019 [20]	2.0	2.0	Multiple time points	23
Clark 2016 [21]	Increase from 35 to 65% of patients with good outcome	Good outcome = 30% improvement over baseline VAS	2 weeks	50

^a^Expected difference between the means of the 2 groups

^b^Calculated number (without losses) reported in publication

The first placebo-controlled trial (Buchbinder in Table 1) estimated “that a sample of 24 participants per group would be required for the study to have 80% power to show at least a 2.5-unit advantage of vertebroplasty over placebo with respect to pain, with a standard deviation of 3.0, based on a two-sided type 1 error rate of 5%” [17]. This is implausible. Medical or surgical treatments cannot be proven effective, useless or harmful by an evaluation of such small number of interventions. Typically, hundreds if not thousands of patients are necessary (see Table 2). This particular study found no difference in a total of 78 patients and concluded that vertebroplasty, a procedure which had by that time been performed in hundreds of thousands of patients, was no more effective than placebo [17]. We fear that the results of such a small trial are at risk of falsely claiming evidence of absence of a treatment effect, the very scenario this calculation was supposed to prevent.

**Table 2:** Sizes of clinically meaningful trials

Lowest risk	Absolute diff	Alpha error	Beta error	Example	Number of patients
5%	5%	5%	20%	Mortality	948
50%	10%	5%	10%	Stroke	816
16.4%	4.6%	5%	15%	COURAGE [22]	2340

The small trial problem is a consequence of the formula used to compare two means. This involves E/S, the expected effect size (E) divided by the standard deviation (S), which is an index of the variability around the mean value of the variable. In the above example E/S = 2.5/3.0 = 0.83, which requires (according to *T* statistics) only 24 patients per group. In the same issue of the New England Journal of Medicine, another vertebroplasty trial (Kallmes in Table 1) used E/S = 1.5/2.7 = 0.56 (which required 65 patients per group) [18]. Interestingly, in that paper, the abstract also reported a post-hoc analysis that used a per-patient criterion of success: a higher rate of clinically meaningful improvement in pain (30% decrease from baseline at the patient level) in the vertebroplasty group as compared to sham (64% vs. 48%, *P* = 0.06) [18]. Looking at the proportion of patients experiencing improvement at 1 month, the previous paper by Buchbinder is so small that had one patient been moved from the “worse” to the “no change” category, statistical significance would have changed [17].

The sole vertebroplasty trial (Clark in Table 1) that used a comparison of the proportions of patients reaching an individual patient outcome threshold to calculate a sample size of 50 patients/group hypothesized an enormous difference (from 35 to 65%) [21]. Yet, that trial showed vertebroplasty to be superior to placebo. The point we want to make is that all vertebroplasty trials were so small they could easily have missed a large difference in the proportion of patients having a better outcome (as large as 20%). Indeed, the largest study, VERTOS IV (Firanescu in Table 1), showed that at 12-month follow-up, a significantly higher percentage of sham patients (41%; *n* = 30) had pain scores of ≥ 5 (on a scale of 10) compared to vertebroplasty patients (20%; *n* = 16): (*χ*^2^ = 8.08, *P* = 0.005, odds ratio 0.36, 95% confidence interval 0.17 to 0.74) [19]. However, this was only a post hoc analysis. The primary outcome showed no difference in mean pain relief (mean VAS) at multiple time points.

The small trial problem is not unique to vertebroplasty studies. A recent trial on lumbar discectomy published in the NEJM similarly proposed that “a sample size of 15 patients in each trial group was calculated for the primary outcome on the basis of an alpha level of 0.05, a beta level of 0.80, a standard deviation of 1.9, and a minimal clinically important difference of 2 on the pain scale” [23]. Another trial on meniscectomy calculated that 40 patients per group would suffice to determine the value of a very frequently performed orthopedic intervention [24].

Trial size estimates should consider the diversity of patients in practice, not only the dispersion around the mean of the selected variable observed in the small number of patients that were recruited. What we propose would require dichotomization of the variable at the individual patient level, which is at first sight contrary to standard statistical recommendations.

## Dichotomizing continuous variables is inadvisable

Good statistical practice is to respect the nature of the variable under study. At the time of analyses, continuous variables should be examined using regression, because dichotomizing continuous variables leads to information loss. Furthermore, dichotomization produces statistical aberrations in multivariate analyzes and potentially results in misleading conclusions [9, 10]. These important problems are beyond the scope of this article. Dichotomization is inadvisable when the focus of the scientific investigation concerns a potential association between a risk factor and patient outcomes. However, this kind of association is weak, typically too weak to serve as a justification for medical interventions. This is reminiscent of the difference in strength necessary to discover a risk factor and the strength necessary to use the same risk factor as a screening test in practice [25].

The method we criticize is derived from Gosset’s seminal work [26]. It is a time-honored method that has been extremely fertile in practical applications. However, when applied to calculate the number of patients required for the trial to be meaningful, the method may yield implausibly small trials. What went wrong?

There are many implicit assumptions when trial size is calculated according to the dispersion of a continuous variable. First, it is assumed that the few data points that you can obtain in such small trials are a representative sample of an imaginary population and that the variable you measured is “normally distributed.” The misleading term “population” seems to refer to patients, but these are populations of variables, since the small group of patients is supposed to be representative of an infinite number of different “populations,” one for each variable. It is also assumed that one patient gaining five points on the scale is equivalent to five patients losing one point or that gaining 1 or 5 points anywhere on the scale has the same meaning no matter where patients or points are on the spectrum. These assumptions are not plausible for most problems of clinical medicine (i.e., glycemia, blood pressure, or most medical variables). In the case of placebo-controlled surgical trials, the variable selected as the primary outcome measure is truly relevant to patients: pain is subjective, but it has been “physicalized” or transformed into a measuring device that gives an impression of pseudo-precision regarding the “quantity” being measured. Pain has been transformed into centimeters on a visual analog scale. Each assumption and transformation adds more uncertainty, rendering trial size calculations even more dubious.

## Clinicians care for patients, not variables

The primary focus of the clinical trial is not the variable, but the treatment being investigated. The clinician does not care for mean population effects but for patients. The targeted treatment must be repeated a sufficient number of times in diverse patients for the evaluation to be clinically meaningful. The variable is only a means to judge clinical outcomes at the individual patient level.

Thus, clinicians need to dichotomize or categorize continuous variables, even though this is usually considered a loss of statistical information. Clinicians must act in practice; they have no choice but to either admit (or refuse admission of) the patient to the hospital or intensive care unit, to operate on the patient or not, etc.… [8] To do so, they need to categorize patients according to potential clinical interventions, which is a very different purpose than categorizing variables for risk factor analyses. The way to establish the thresholds that will be used to categorize a patient as normal or not, or a treatment as successful or not, is a difficult problem beyond the scope of this article. It is the necessity of action in clinical practice which requires a judgment to be made at the individual patient level. The clinician needs trial results to be meaningful in terms of patients, just as a diagnostic study needs a threshold at the individual patient level to sort out patients who should be operated on from those who should not.

Similarly, the clinically meaningful difference that must be estimated to calculate trial size is the one that makes a difference for individual patients. Moreover, and contrary to studies of exposures to risk factors and etiology of diseases at the level of populations, clinical trials that assess medical interventions concern individual patients, each with their own pain experience, treated using interventions performed one patient at a time.

The different ways that continuous variables are treated and used depend on the priority and objectives of the study, whether it is designed to explain or understand the mechanisms of treatment, or to inform clinical practice. If continuous variables should be left alone at the time of risk factor analyses, comparing the means of continuous variables may not be the proper instrument to measure the value of a treatment in clinical care. In clinical practice, the indivisible unit is the patient. For clinical purposes what counts is the diversity or heterogeneity of patients, holistically considered individuals that differ in many more respects than the variability revealed by the standard deviation of the variable that was selected as the primary outcome in order to minimize trial size.

We cannot provide a rule that would prescribe a minimal number of patients that should be recruited in a particular trial, but a general idea of the sizes of trials that result from using a comparison of proportions of patients reaching a per-patient outcome of interest is offered in Table 2.

## Is the trial testing the treatment or the accuracy of the measuring instrument?

One further example offers additional support to this thesis. ORBITA, another placebo-controlled trial, was designed to test the value of coronary stenting in the management of angina caused by severe stenosis of a single coronary artery. The size of the trial was calculated using time on the treadmill test, a continuous variable measured in seconds. ORBITA showed that 105 patients allocated stenting outperformed 95 patients allocated the placebo intervention by a non-significant mean of 16 seconds on the treadmill [27]. This result was interpreted as showing that stenting was no more effective than placebo. Another interpretation may be that ORBITA proved the poor diagnostic accuracy of the treadmill test to reliably distinguish patients with or without severe coronary artery stenosis. A different approach is necessary to condemn a routinely performed clinical intervention to obsolescence. In the case of coronary stenting, a more convincing study was the COURAGE trial which examined a more patient-relevant, per-individual clinical endpoint (death or non-fatal myocardial infarction), in a more meaningful number of patients (*n* = 2287) [22].

If the trial is to inform practice, the experimental treatment must be tested against controls in a meaningful number of repetitions, in a diversity of patients. The important idea behind planning the size of the trial is the recruitment of a sufficient number of patients to determine good medical practice.

## The primacy of patients

The problem we have examined is reminiscent of the famous medieval debate regarding “Aristotelian universals”: should the properties belonging to an object, that the object has in common with other objects, be considered to exist beyond those objects, in and of itself? In our problem, do variables, risk factors, and pain or function scales exist by themselves detached from patients? The solution we propose resembles the position attributed to Occam, who is legendary for the admonishment to not multiply entities without necessity [28, 29]. The fundamental entity of interest in medical care is not the numerical value, distribution or dispersion of a variable, attribute, or property in a population but the individual patient.

## Conclusion

A meaningful trial is a trial that has included a sufficient number of individuals belonging to a sufficiently wide spectrum of patients for conclusions to safely apply to the diversity of patients that will come to medical attention in the future. In general, trials designed to inform practice should be large and the number of patients to be recruited should be estimated using the comparison of proportions of patients reaching a clinically significant individual patient outcome.

## Data Availability

Not applicable.
